# New-Onset Seizure as the Only Presentation in a Child With COVID-19

**DOI:** 10.7759/cureus.8820

**Published:** 2020-06-25

**Authors:** Sabita Bhatta, Abida Sayed, Bandana Ranabhat, Raj Kumar Bhatta, Yogesh Acharya

**Affiliations:** 1 Pediatrics, Woodhull Medical Center, New York, USA; 2 Medicine and Research, Avalon University School of Medicine, Willemstad, CUW; 3 Pediatrics, College of Medical Sciences, Bharatpur, Chitwan, NPL; 4 Neurosurgery, Postgraduate Institute of Medical Education and Research, Chandigarh, IND; 5 Vascular and Endovascular Surgery, Western Vascular Institute, Galway, IRL

**Keywords:** 2019 novel coronavirus disease, covid-19, seizures, afebrile, pediatrics, children, clinical presentation

## Abstract

We present a child with a new-onset isolated afebrile seizure in coronavirus disease 2019 (COVID-19). This patient, an 11-year-old Hispanic male, was brought to our ED in New York city on May 01, 2020, during the ongoing COVID-19 crisis with seizure. There was no fever and/or respiratory and gastrointestinal complaints. His general and systemic examination did not reveal any abnormality. Similarly, his biochemical profiles were within normal limits, and the radiological study, including a chest X-ray and CT scan, showed normal findings. His polymerase chain reaction (PCR) was positive for SARS-CoV2. The patient was admitted for observation after consultation with pediatric neurology, and his condition progressively improved with anti-seizure medications. This case highlights the need for recognizing an uncommon and atypical presentation in COVID-19 as the new cases are unfolding rapidly across the globe.

## Introduction

The novel coronavirus disease 2019 (COVID-19) has impacted thousands of children worldwide [1]. Despite being generally mild in children, we have witnessed vague clinical pictures in COVID-19, ranging from asymptomatic in the mildest form to severe respiratory distress [2]. Recently, there have been multiple reports of atypical symptoms in children, necessitating recognition of uncommon disease presentations to protect the vulnerable populations, and minimize complications [3- 4]. Neurological manifestations in children have not been widely reported. Here, we aim to report an uncommon neurological manifestation, isolated afebrile seizure, in a child with COVID-19.

## Case presentation

An 11-year-old Hispanic male child was brought to our ED with abnormal shakiness of the body that lasted one to two minutes. His mother described the event as sudden shakiness of the whole body, associated with stretching and tightening of all four limbs, uprolling of eyes, frothing from the mouth, and tongue bite without urinary or bowel incontinence. He recovered slowly and started recognizing and talking to his mother after 10-15 minutes following the event. The patient was generally well, and there were no other complaints, including fever or shortness of breath. There was no significant past medical or surgical history, and his birth was uneventful. His immunization status was up-to-date, and he achieved all his age-appropriate developmental milestones. 

During his ER visit in our hospital, he had another generalized tonic-clonic seizure that lasted for two to three minutes. There was generalized jerking movement of the whole body associated with abnormal tightening of both upper and lower limb, uprolling of the eye, and clenching of teeth. The seizure was terminated using lorazepam 2 mg intravenous stat. 

On examination, the patient was well-appearing and interactive. He was alert and oriented to time, place, and person. His temperature was 99℉, respiratory rate 18 breaths/min, blood pressure 115/70 mmHg, heart rate 90 beats/min, and oxygen saturation 95%. There was a bite mark in the tongue with minimal bleeding. The head ear nose throat (HEENT) examination revealed normo-cephalic and atraumatic head with b/l equal and reactive pupils, and normal conjunctiva. The neck was supple with a complete range of motion. His nervous system examination showed intact cranial nerves (CN I-XII), normal motor examination with normal bulk and power, normal deep tendon reflexes, intact sensation, and no cerebellar signs. Cardiovascular examination showed a normal rate, regular rhythm, normal heart sounds, and no gallop or murmur signs. Pulmonary/chest examination revealed no signs of respiratory distress, wheezing, or rales. Abdominal examination revealed normal bowel sounds with no signs of distention and tenderness. His skin was warm and dry without diaphoresis and erythema. Examination of the musculoskeletal system displayed a normal range of joint motion with absent edema and tenderness. 

**Figure 1:**
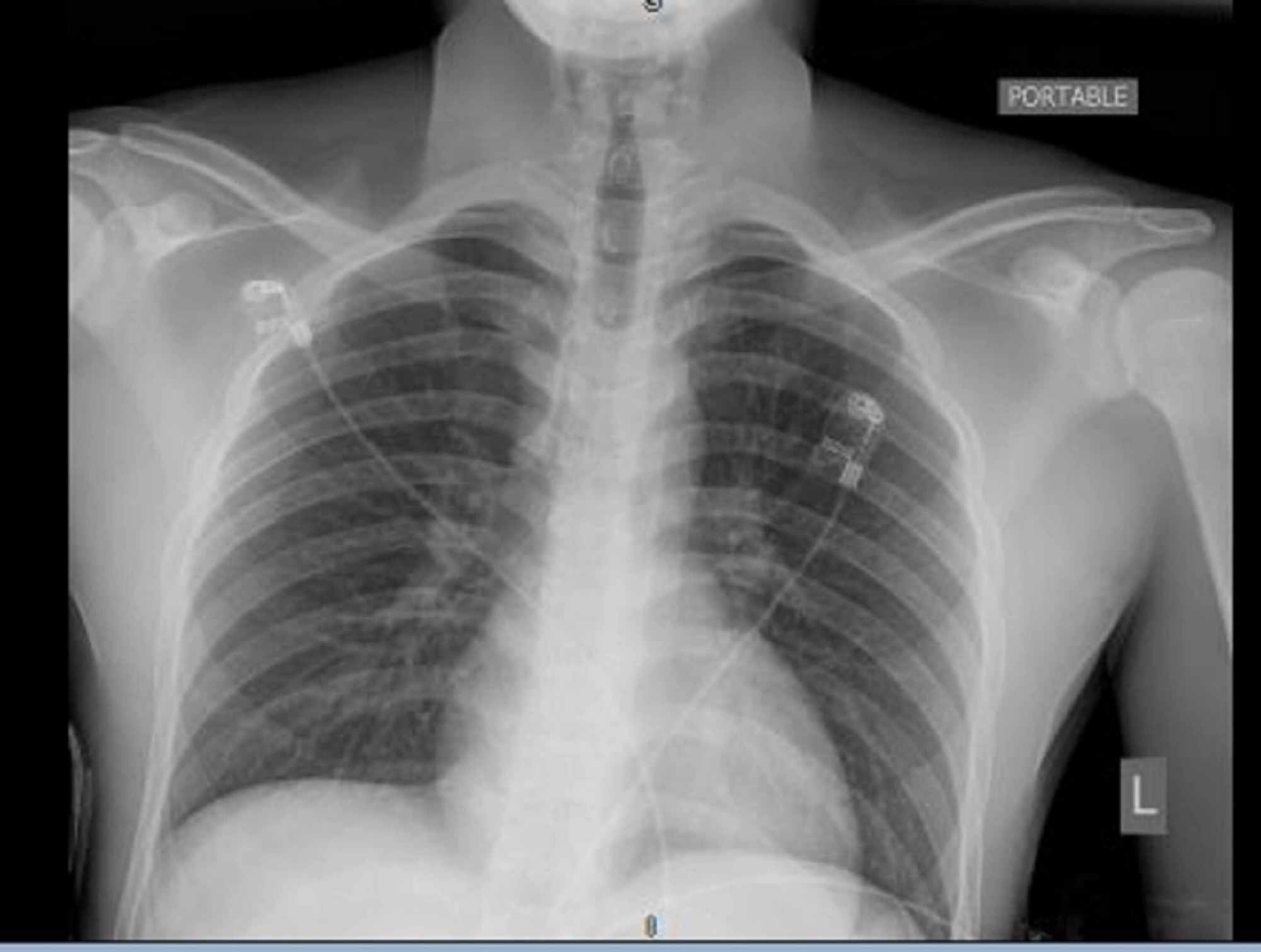
Portable chest X-ray on admission revealing no abnormal signs.

**Figure 2:**
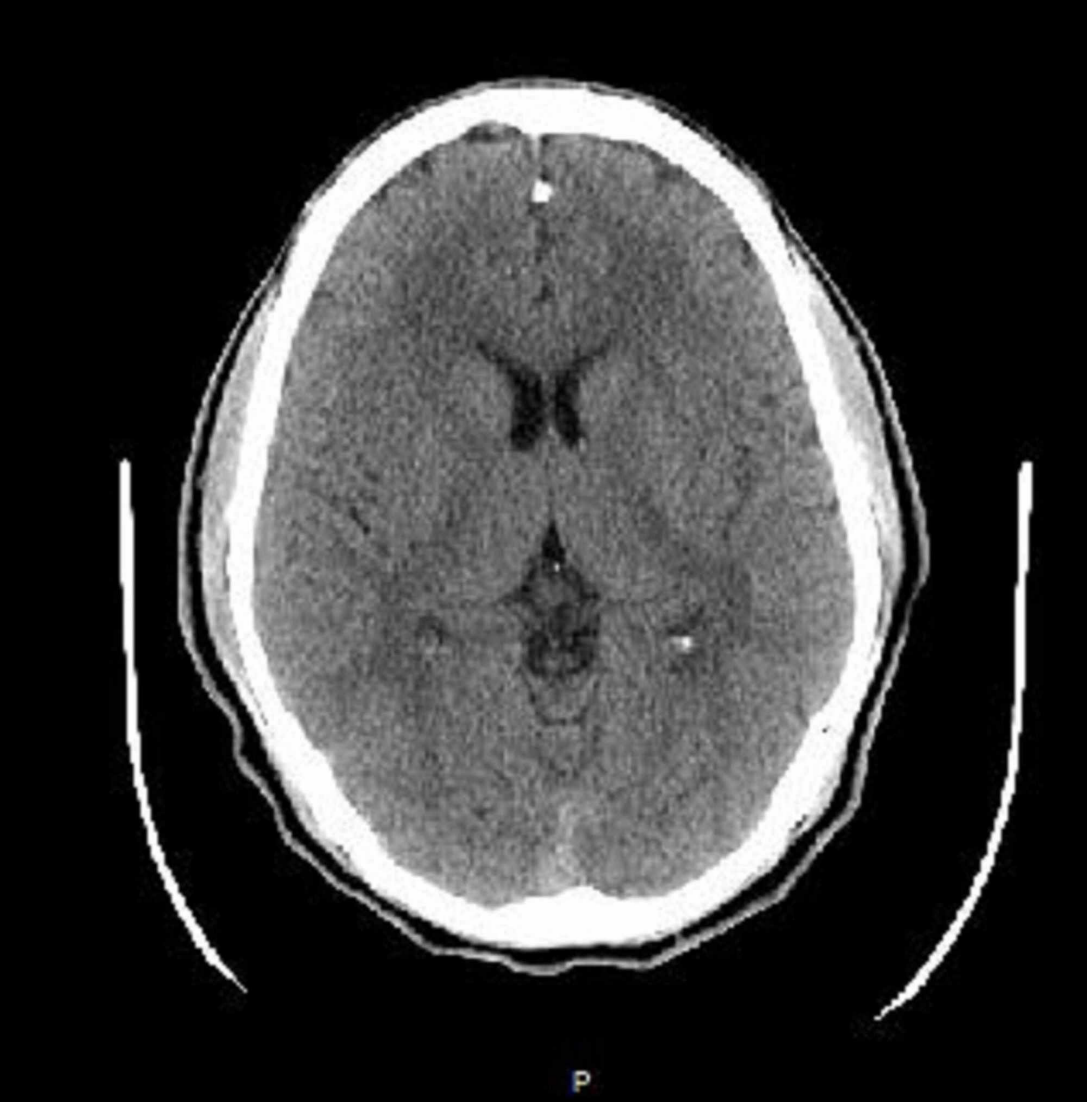
Head CT scan without contrast on admission showing no abnormal features.

As the patient belonged to the current COVID-19 endemic area, his new-onset seizure was suspected to be triggered by COVID 19 infection. Laboratory and radiological investigations were planned to support the diagnosis and rule out other serious causes of seizures in children, including meningitis, encephalitis, hypoglycemia, dyselectrolytemia, and substance abuse. Laboratory findings showed white blood cells (WBC) 8.5 cells/mm3, and comprehensive metabolic panel (CMP) with Na 137 mEq/L and K 4.5 mEq/L. His coagulation profile, C-reactive protein (CRP), procalcitonin, ferritin, and d-dimer were within normal limits. Blood toxicology, including the drug panel, was negative. There were no abnormalities in the chest X-ray and brain CT (Figures 1-2). His viral polymerase chain reaction (PCR) test for the severe acute respiratory syndrome coronavirus 2 (SARS-CoV-2) ribonucleic acid (RNA) was positive; however, the test for influenza was negative.

We consulted with the pediatric neurology department and decided to admit the patient for observation. The electroencephalogram (EEG) was planned only if a patient had clinical deterioration. He was managed with levetiracetam 500 mg twice daily. During the in-ward hospital stay, the patient was stable and did not have seizures or other complaints. He was discharged the next day and scheduled for a follow-up in a week. During his follow-up, the patient looked fine and reported neither seizure nor any complaints in the preceding week. 

## Discussion

The majority of the children with COVID-19 are either asymptomatic or present with mild symptoms when compared to adults [5]. Fever, cough, and gastrointestinal symptoms are common; however, many atypical presentations have been reported recently [2]. Description of isolated afebrile seizure in COVID-19 is scarce among children, and neurological manifestations have not been extensively studied [6-7]. 

Our case highlights seizure as one of the uncommon, but potential presentations of COVID-19 in children. Following nasal infection, the SARS CoV-2 can enter the brain via olfactory bulbs, resulting in inflammation and myelin damage. Mao et al. evaluated 214 COVID-confirmed patients for neurological manifestations [8]. Their findings revealed central nervous system manifestations in one-fourth of the patients: dizziness and headache being the most common presentation. The central nervous system's involvement in COVID-19 can cause headaches, dizziness, altered consciousness, ataxia, seizures, and even encephalitis and meningitis in severe form. 

In pediatric age groups, the neurological complications have not been widely reported [9]. But as the new cases are unfolding every day, we cannot ignore the possibilities of atypical symptoms and/or variable presentations. Musolino et al. investigated preliminary COVID-19 findings and found one out of 10 infected children with seizures, while others presented predominantly with fever, cough, and diarrhea [10]. Subsequently, Dugue et al. reported seizures in COVID-positive infants. However, this patient also had a fever and mild hypertension, along with a history of siblings with streptococcal pharyngitis [11]. The fit was associated with a sustained upward gaze, bilateral leg stiffening, and reduced responsiveness. The SARS-CoV-2 RNA and rhinovirus c sequences in swab samples (nasopharyngeal/anal) were positive in this particular patient, confirming a co-infection. Seizures are generally common in viral infections, particularly with adenovirus, influenza, rhinovirus, and respiratory syncytial virus (RSV) in children 12 years and younger [11]. Coronavirus in infants can be associated with a 27% chance of a co-infection with rhinovirus, and there is a possibility of other co-infection [12-13]. 

In our case, the patient did not have any respiratory or gastrointestinal symptoms. However, viral PCR tested positive for COVID-19. We admitted the patient for observation/isolation and subsequently ruled out more severe seizure etiologies, like hypoglycemia, dyselectrolytemia, and infections. Although we could not trace the potential source of COVID-19 infection in the patient's family, we can assume that the patient contracted the disease owing to a recent surge in COVID-19 cases in his residential area across New York City, USA. 

This case underlines the uncommon neurological manifestation, seizure, triggered by COVID-19. Seizure in children can have serious consequences, including physical damages; therefore, it is crucial to recognize the potential causes and manage them without delay. There is a necessity for general guidelines that incorporate detailed clinical investigations with a neurological examination in pediatric patients, especially from endemic areas, to rule out any severe neurological sequelae of COVID-19 in this pandemic. 

## Conclusions

Our case describes a new-onset isolated afebrile seizure in a child with COVID-19. As new cases are unfolding each day, it is essential to recognize seizure as a potential COVID-19 presentation in the pediatric age groups. Neurological involvement can lead to severe complications and long-term sequelae. Therefore, we recommend physicians to rule out neurological involvement in all children with COVID-19.
